# Plus Disease: Why is it Important in Retinopathy of Prematurity?

**DOI:** 10.4103/0974-9233.63080

**Published:** 2010

**Authors:** Carlos E. Solarte, Abdulaziz H. Awad, Clare M. Wilson, Anna Ells

**Affiliations:** Division of Pediatric Ophthalmology, King Khaled Eye Specialist Hospital, Riyadh, Kingdom of Saudi Arabia; 1 Department of Optometry and Visual Science, UCL Institute of Ophthalmology, Northampton Square, London EC1U0HB, UK; 2 Calgary Retinal Consultants, Calgary, AB, Canada

**Keywords:** Diagnosis, Plus Disease, Prematurity, Pre-Plus, Retinal Detachment, ROP, Treatment

## Abstract

Retinopathy of prematurity (ROP) is one of the leading causes of preventable blindness in childhood. Early posterior pole vascular signs of severe ROP have been studied since the first description of the disease. The progressive changes that take place in the posterior pole vessels of an extremely premature baby occur in a predictable fashion soon after birth. These vascular changes are described as plus disease and are defined as abnormal dilation and tortousity of the blood vessels during ROP that may go on to total retinal detachment. The ophthalmological community now has a better understanding of the pathology and cascade of events taking place in the posterior pole of an eye with active ROP. Despite many years of scientific work on plus disease, there continue to be many challenges in defining the severity and quantification of the vascular changes. It is believed that understanding of the vascular phenomenons in patients with ROP will help in designing new treatment strategies that will help in salvaging many of the eyes with severe ROP.

## INTRODUCTION

Retinopathy of prematurity (ROP) results from important changes occurring in the posterior retinal vessels of eyes of pre-mature infants. Since the initial classification of retrolental fibroplasia in 1953, many attempts have been made to identify those eyes that may progress to retinal detachment and blindness if not treated in a timely fashion.^1^ Activity in the posterior vascular architecture indicates significant level of severity of the disease. Some reports have described vascular changes including tortuous arterioles and venules that tripling in size as part of the active and progressive course of ROP. In 1982, Quinn *et al.*^2^ coined the term *“Retinopathy of Prematurity Plus*” to describe a virulent form of ROP. Their work defined the differentiation between two clinical forms of ROP which differed in prognosis and potential timing of the treatment. The international classification of ROP described in 1984 and its revision in 2005 used the term plus disease to signify the vascular features of ROP that may alert the attending ophthalmologist to for consider treatment of the premature avascular retina. The purpose of this review is to describe the current understanding of the clinical presentation, pathophysiology, value of diagnostic tools, and discuss treatment options in the management of patients with ROP/plus disease.^3^,^4^

## PATHOPHYSIOLOGY

There are many studies focused on the potential etiologic factors contributing to ROP. The introduction of pulmonary oxygen therapy for preterm infants in the 1940s, played a major role in an epidemic of ROP that subsequently occurred.^2^ After more than 50 years of continuous research and over 4000 published articles, there is a better understanding that ROP is not just due to a single cause but is due to a combination of additional multiple external factors. Current concepts of pathogenesis of ROP have evolved from basic science research by observing events at a cellular level as well as from several animal models. Among the various theories, the “classical” and the “gap junction theory” are currently the two major theories on the development of ROP. The “classical” theory proposed by Patz^3^ and Ashton^4^ describes an initial hyperoxic phase of the disease which causes arteriolar constriction with subsequent irreversible vaso-obliteration. This is then followed by a second phase in which a vaso-proliferative response is induced by retinal ischemia as a result of retinal capillary closure. The “classical” theory has been followed by the “gap junction theory” of Kretzer and Hittner.^5^ Their theory of pathogenesis is based on the activity of mesenchymal spindle cell precursors of retinal capillaries. Accordingly, these cells migrate centrifugally from the optic disc toward the junction between vascular and nonvascularized retina, to form a new capillary network. Under hyperoxic conditions, abnormal gap junctions appear between adjacent spindle cells, and this interferes with normal cellular migration and vascular formation. The angiogenic factors secreted by these mesenchymal cells may in turn trigger a neovascular response.^6^,^7^

Mesenchymal cells (such a primitive astrocytes), of the ischemic, nonvascularized peripheral retina produce vascular endothelial growth factor (VEGF) which in the absence of any regulation, stimulate the production of neovascularization in ROP.^8^,^9^ Under normal conditions, the presence of VEGF in response to physiological hypoxia of the maturing avascular retina just anterior to the junction with vascular retina, support normal angiogenesis. It has been proven that VEGF may be highly sensitive to hyperoxia–hypoxia changes.^10^ Insulin-like growth factor 1 (IGF-1) has been described as a regulatory factor for VEGF under physiological and abnormal conditions. This factor is produced between second and third trimester by the placenta, and it is oxygen-independent. IGF-1 participates in the regulation of VEGF within the retina. Its absence due to premature birth and loss of the placenta plays a major role in the pathogenesis of ROP, because VEGF is then produced by the ischemic retina without any regulatory factors, resulting in the abnormal angiogenesis characteristic of ROP.

The premature retina responds dramatically to the presence of VEGF. The presence of VEGF results in the remodeling of vessels as a direct response to fluctuations in oxygen from an ischemic bed.^2^,^11^,^12^ As a consequence of the effect of VEGF, there is an important increase in blood flow with related changes in vessels shape.^2^,^11^–^13^ Such changes are not exclusively due to the presence of VEGF but also occur as a result of flow mechanics. Venules are distensible and arterioles are generally not. Hence the initial response of the venules is dilation whereas the arterioles becomes tortuous.^2^ These vascular changes develop over weeks of chronological age in a premature infant and usually mild venous dilation occurs first, followed by arteriolar tortuosity at the posterior pole and posterior vascularized retina.^2^,^14^

**Figure 1:**
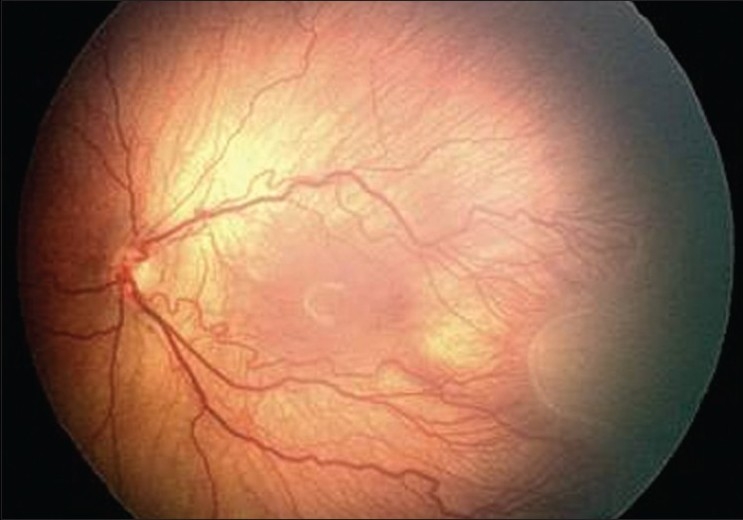
Image the posterior pole with presence of pre-plus and ROP seen in the temporal quadrants

Different mechanisms have been proposed to explain the cascade of vascular events taking place in the posterior pole in the presence of ROP. Arteriovenous shunts develop because of the reduced capillary resistance from the remodeling of cells combined with an increase in retinal blood flow.^1^,^8^ During the hypoxic phase of the disease, impaired smooth muscle cell differentiation may be responsible for an inability to regulate blood flow.^15^ Other studies have proposed that VEGF may be present in the vitreous of an eye acting directly on central retinal blood vessels causing dilation and tortuosity.^13^ This may explain in part why vascular changes in the posterior pole of retina appear prior to neovascularization in cases of severe ROP.^2^ The vascular changes seen in the posterior pole consisting of dilated venules and tortuous arterioles in the presence of severe ROP are known as *plus* disease.^1^ Vascular changes that are not normal, but are insufficient for the diagnosis of plus disease are clinically defined as *pre-plus* changes of ROP.^16^ The term “pre-plus” was introduced in the revision of the classification system in 2005, to help the attending physician aware of the presence of a vascular abnormality although the severity was not in keeping with the significant changes known as plus disease [Figure 1].

## DIAGNOSIS AND QUANTIFICATION OF PLUS DISEASE

A wide spectrum of posterior retinal vascular changes exists in ROP. Plus disease describes the most severe vascular changes of dilation and tortuosity and is associated with severe ROP and visual morbidity if left untreated. The diagnosis of plus disease is historically a dichotomous decision based on subjective comparison to reference images.^1^,^17^ Since the introduction of the term pre-plus, there has been much debate surrounding the accuracy of clinician differentiation between plus disease and pre-plus disease.^2^,^18^ Plus disease is challenging to diagnose, differing among clinicians partly as the examination is qualitative and subjective, and partly due to the imperfect and difficult task of examining a moving and sometimes poorly dilated eye of a premature infant.^2^ The qualitative evaluation performed during indirect ophthalmoscopy remains contentious among the most expert observers, and many attempts have been made at standardization and quantification.^19^,^20^ However there is geneal acceptance that the presence of plus disease is a marker for severe disease, and it is one of the most important prognostic indicators in ROP.^16^,^21^,^22^

According to current protocols and international standards for screening for ROP, all infants with a birth weight less than 1500 g and/or less than 32 weeks of gestational age, are required to have weekly bedside retinal examination by a specialist with experience in ROP.^21^–^24^ During this screening, a complete evaluation must be performed. The stage or severity of the disease is recorded according to the International Classification of Retinopathy of Prematurity (ICROP), in addition to the zone of the disease and the presence or absence of pre-plus or plus disease.^16^ This internationally accepted classification of the morphological description of ROP allows global standardization of diagnosis and management of the disease. There is no absolute time in which the clinical appearance of plus disease can be regularly detected at bedside, although typically it develops between 34 and 38 weeks of post-menstrual age, depending on the gestational age of the infant.^25^ The lower the gestational age, the earlier plus disease may be expected to develop.^21^,^22^ Plus disease develops in the same time frame as the other clinical features of ROP, such as an intraretinal ridge of mesenchymal tissue (stage 2), or neovascularization (stage 3). Wallace *et al*.,^26^ Saunders *et al*.,^11^ and Freedman *et al*.^27^ report an early dilation and tortuosity as insufficient for plus disease diagnosis or pre-plus vascular changes as early as 33 weeks postmenstrual age. Pre-plus as well as plus vascular changes, therefore, have prognostic significance in the early course of ROP.^26^

**Figure 2:**
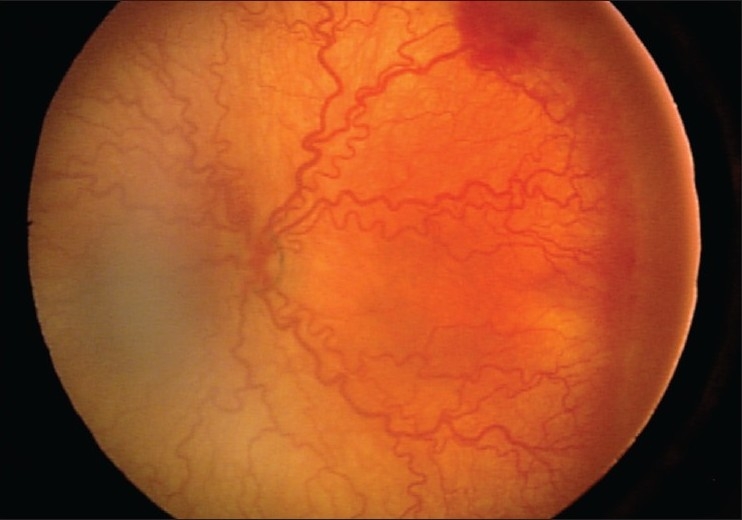
Presence of severe aggressive posterior ROP with intense plus and minimum changes in the periphery

Apart from the classic ROP presentation which is fairly predictable in timing of progression and regression, there is a newly recognized form of ROP called “aggressive posterior ROP” (AP-ROP). AP-ROP represents the most severe active form of the disease. AP-ROP occurs in the youngest and smallest infants and does not progress through the stages of severity of disease as in the classic form. AP-ROP is posterior in location and may occur in zone I or the posterior aspect of zone II. Plus disease develops early and is pronounced in all four quadrants. Often there is no ridge at the junction between vascularized and nonvascularized retina and only a flat network of neovascularization may be present. This can be difficult to view using indirect ophthalmoscopy; however, the massive, early and unusual presence of plus disease may be a major marker of this virulent form of ROP^13^ [Figure 2].

Recent research has addressed potential quantitative approaches to the diagnosis of vascular changes in ROP. There is disagreement among experts on both the diagnosis of pre-plus and plus disease showing discrepancies among observers.^2^,^16^,^28^,^29^ There are many possible advantages of the use of a standardized, quantitative classification system for vessel changes of the posterior pole in ROP. First, the use of plus as a marker for the development of severe ROP or ET-ROP Type I ROP that may require treatment.^26^,^30^ Second, when examination of the periphery cannot be accurately assessed due to instability of an infant or poor dilation, the presence of plus disease can lead to the diagnosis of severe ROP.^11^,^26^ A quantitative classification system may also have potential applications in clinical research such as early laser treatment of severe ROP,^26^ or when the screening evaluation necessitates a telemedicine approach.^28^,^31^,^32^

The posterior pole changes may be more easily seen than zone or stage of ROP.^19^,^26^ In an attempt to quantify plus disease, several methods including clinical and semi-automated methods have been described.^2^,^33^ Saunders *et al.* to our knowledge were the first group to attempt a grading system for the vessel changes of ROP,^11^ introducing a clinical grading system ranging from 0 to 4. Wallace *et al.*^26^ modified the grading system including pre-plus and realized its relation with the prognostic significance in the early course of ROP.^2^,^32^ Wilson *et al.*^33^ present a novel technique to quantify vessel morphology grading on a likert scale for both width and tortuosity. It may be the first report to use a 0–10 scale to quantify the retinal vessels on high-quality digital images of infants with and without ROP. In 139 images from preterm infants at risk of developing ROP, five expert clinicians, three junior ophthalmologists, a neonatal nurse, and an orthoptist graded the largest venule and the largest arteriole in each quadrant on a 0– 10 scale for width and tortuosity. The novel vessel grading system has so far shown a high degree of sensitivity for arteriolar readings at detecting both peripheral stage 3 and ROP requiring treatment. Interestingly, arteriolar grading show a higher degree of sensitivity than other vessels parameters and plus diseases status (PDS).^34^ This clinical study quantifying the vessel changes of ROP has been used as the basis for the most recent development of automated software in the same group.

**Figure 3:**
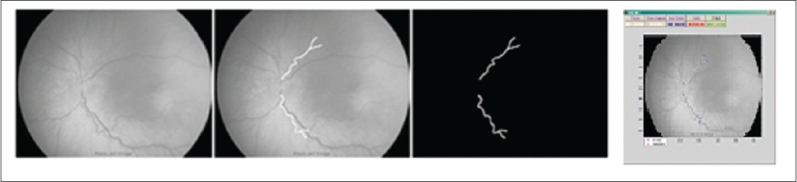
Four of the stages of RISA vessel analysis. Virgin image, skeletonisation of vessels, and finally the produced binary image

As explained, the inconsistencies and subsequent anxieties placed on the ROP examination could be alleviated by a quantifiable system of ROP screening, potentially to be performed automatically by computer image analysis tools. One of the initial semi-automated programs available for assessing the retinal vessel changes in preterm infants was developed by Martinez-Perez at Imperial College London.^35^ This program uses vessel segmentation based on the first and second derivatives of the intensity of the image, maximum gradient, and principal curvature. Region growing techniques are then employed to progressively segment the vessels using information about the surrounding eight neighboring pixels [Figure 3]. The system takes approximately 20 min for analysis and has been used to quantify the retinal vessel width and tortuosity in research settings.^36^,^37^

**Figure 4:**
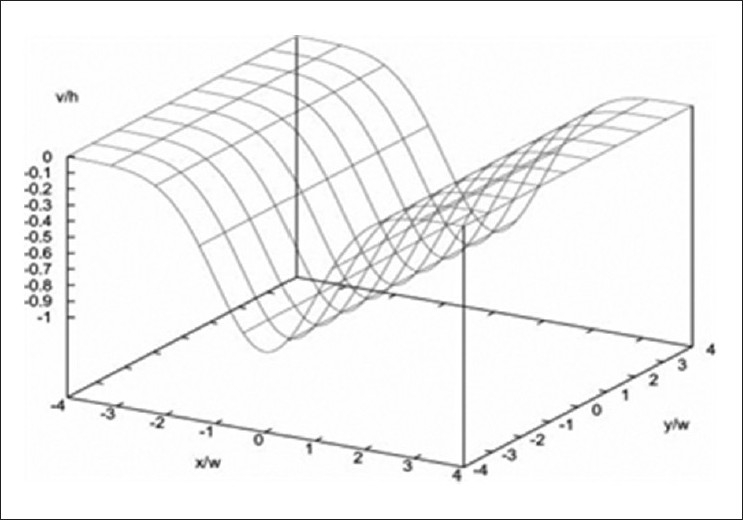
Illustration of estimated model of Gaussian profile with parameters of height, width, and orientation computed at each location (reproduced from IOVS 2008)

**Figure 5:**
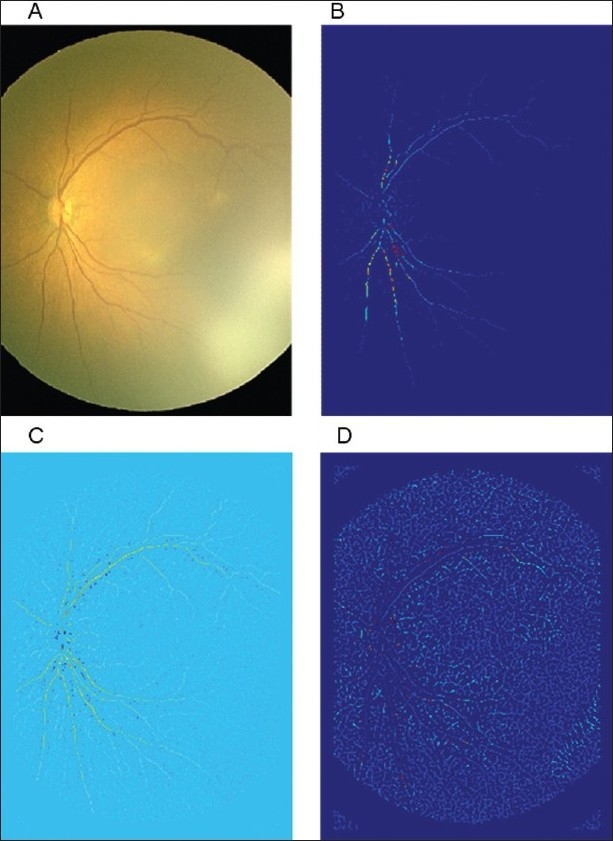
Stages Of CAIAR (a) original RetCam image, (b) height map, (c) isocontrast map (the isovalues of the venules are higher–represented by yellow coloration), and (d) width map

Subsequent to retinal image search and analysis (RISA), computer-aided image analysis of the retina (CAIAR) was developed at Imperial College London. The user input necessary was reduced to helf by more intuitive vessel location abilities based on maximum likelihood model-fitting in a scale space framework. The estimated model of Gaussian profile with parameters of height, width, and orientation are computed at each location in the image as illustrated in Figures 4 and 5.

**Figure 6:**
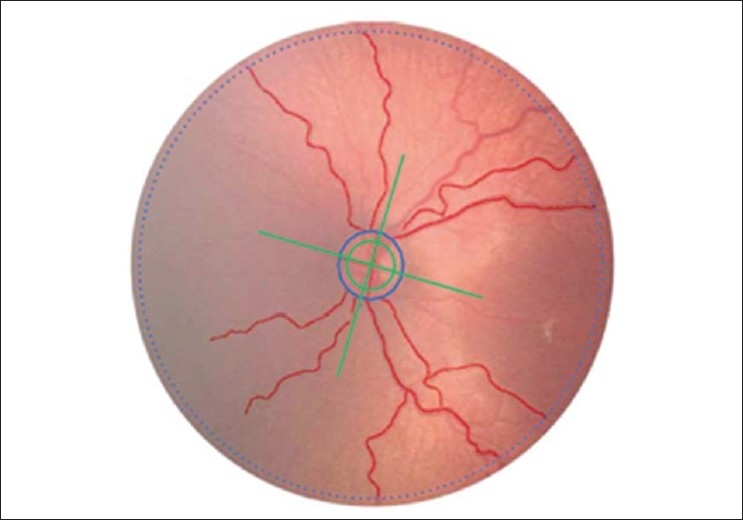
*Solid blue circle* is optic nerve border; *dotted blue circle* is as though viewed through a 28-diopter circle; *green lines* are borders of quadrants; *red lines* are traced vessels whose tortuosity has been calculated

Concurrently, ROPtool has been under development at Dukes University, North Carolina, an extrapolation of a technique for measuring tubular objects in three-dimensional images initially used for locating the intracerebral vasculature from magnetic resonance angiography (MRA) images.^3^,^38^ The user identifies an initial start point of a retinal vessel, and the intensity ridge is automatically located, outlining the vessel [Figure 6].

**Figure 7:**
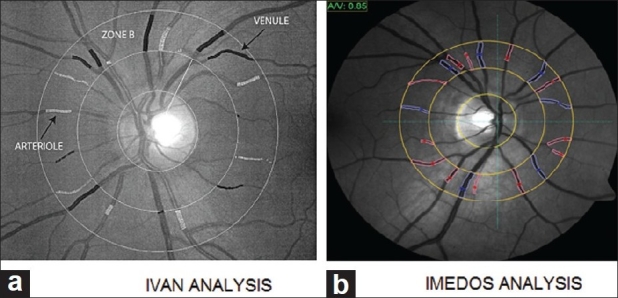
Image undergoing IVAN analysis (a) and Imedos analysis (b)

**Figure 8:**
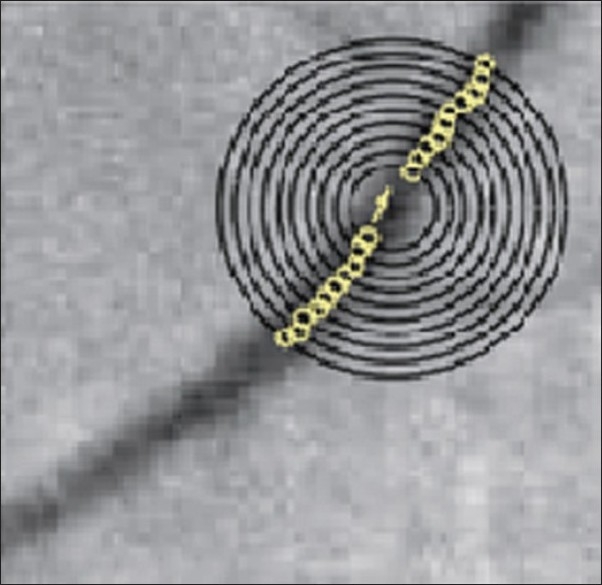
*Black*: the circular scan lines; *white*: the identified vessel centers. The *white arrow* shows the estimated vessel direction

Other programs have been adapted from diabetic retinal image analysis techniques for analysis of images from preterm infants. Vasculomatic a la Nicola (IVAN) is one such program. IVAN measures retinal vessel diameters of the six largest arterioles and six largest venules located 0.5–1.0 disc diameters from the disc margin [Figure 7a]. Each image takes around 20 min of trained user input for analysis. Imedos have developed a similar tool for detecting vessel structure [Figure 7b]. Bubble analysis techniques have been used to detect vessels by a group in Italy [Figure 8].^39^

**Figure 9:**
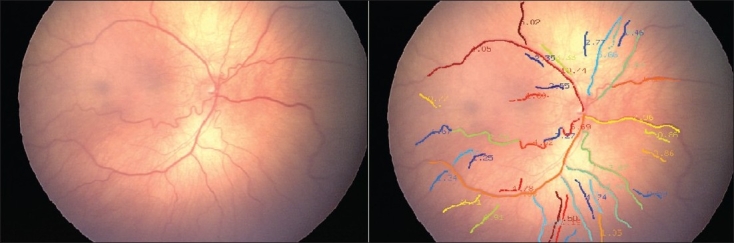
Good quality RetCam image pre and post-RetVas analysis

**Figure 10:**
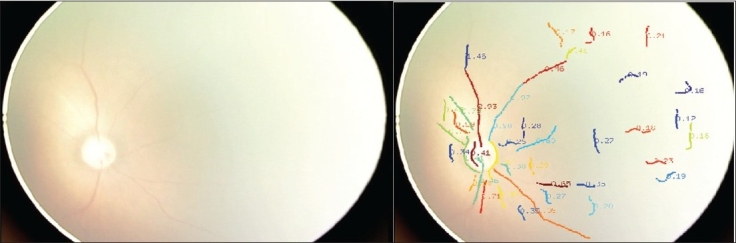
Bad quality RetCam image pre- and post-RetVas analysis

Recently, a completely automated system, RetVas has been developed to detect the retinal blood vessels of images from preterm infants at risk of developing ROP. A sensitivity of 97% for locating one arteriole and one venule in each quadrant of 75 images has been reported. The software is based on a model reverse engineered from human vision, using the same processes as the human visual cortex to observe images and select features. Pre- and postautomated analysis images are illustrated in Figures 9 and 10, showing the ability of RetVas to work with high-quality and low-quality images, as necessary in ROP screening, to provide a quantified result for the width and the tortuosity of the localized vessels^34^

Each vessel localization program uses its own algorithm in calculating the width and or tortuosity of the blood vessel, it has segmented. For example, CAIAR uses a multi-scale approach successively subdividing the vessel sections into two parts. The geometric concept involves perpendicular bisection of the vessel chord at its midpoint and subsequent reapplication of the subdivision on the resultant segments until the segment lengths fall below a specified value (four pixels).^33^ Freedman *et al.*^27^ made use of computer technology to quantify vascular dilation and tortuosity in ROP. Wallace *et al* using the same principle of measuring a series of points along each vessel to calculate vessel tortuosity, Wallace *et al*.^26^ were able to calculate using retinal images of 20 premature infants whether or not an eye had plus disease with a sensitivity of 80% and a specificity of 91%.^26^ Currently, many other techniques and computer software systems are available to measure retinal blood vessels by the use of high-quality Ret-Cam images.^2^,^39^,^41^ ROPtool measures tortuosity by comparing the total length of the vessel to the length of a smooth curve created by the software following several points along the vessel. This has been reported to be a reliable method of measuring tortousity sufficient for the diagnosis of plus disease.^39^

Despite great advancements in the area, there are many barriers to the implementation of new concepts for real-time screening at bedside. Firstly, most of these programs are not fully automated and require a trained operator to select a vessel. Secondly, the degree the accuracy of such a system requires rigorous testing in the form of multicenter clinical trials to ensure the small changes between normal and abnormal vessels are adequately and repeatedly detected in infants with and without ROP. Third, images—specifically high quality images, may not be widely available due to equipment restrictions.^2^

## ROLE OF PLUS IN ROP TREATMENT

The goal of treatment of Type I ROP is to remove the stimulus for abnormal neovascularization due to an ischemic retina.^21^ The most important factor in deciding when treatment may be required is to determine the timing at which point vascular changes may indicate the progression to severe disease and chances of visual outcome remain optimum. Since the publication of the International Classification of ROP in 1984 and its expansion in 1987, with the identification of ROP by location of retinal involvement (such as zone, extent, and stage of the disease), the presence or absence of dilated and tortuous posterior pole vessels (plus disease) has been used to determine the indication for the treatment.^1^,^29^“Threshold” parameter (the stage when the risk of blindness is over 50% if untreated) has been established since these classifications. The results of the Multicenter trial of Cryotherapy for Retinopathy of Prematurity of 1988 found that 45.4% of the treated eyes maintained 20/100 vision or less showing the need to lower this threshold.^22^,^29^

Most recently, the Early Treatment Trial for ROP (ET-ROP) has defined the severity of ROP into two types, Type I and Type II, recommending the guidelines for treatment as outlined in Table 1. Type I severity requires treatment with pan-retinal photocoagulation to the avascular retina within 48 h of diagnosis, while Type II disease can be followed with weekly examinations.^21^ The constant clinical feature in Type I ROP is the presence of plus disease and hence is a clear indication for treatment [Table 2]. Current tendencies in the management of ROP are on favor to perform earlier intervention in eyes defined as “pre-threshold” or with posterior pole changes such as pre-plus or posterior aggressive ROP which does not fall into the classical definition of plus disease but of clinical importance when deciding treatment.^24^,^41^ Pan-retinal photocoagulation is indicated for eyes that present any of these signs. These aspects mark the moment where treatment on the ischemic retina should be applied.^3^,^5^

**Table 1:** Recommended guidelines for the treatment of ROP

Type 1	Zone I, Any stage with Plus
	Zone I, Stage 3 with or without plus disease
	Zone II, Stage 2 or 3 ROP with plus disease
Type 2	Zone I, Stage 1 or 2 without plus
	Zone II, stage 3 ROP without plus disease

**Table 2:**
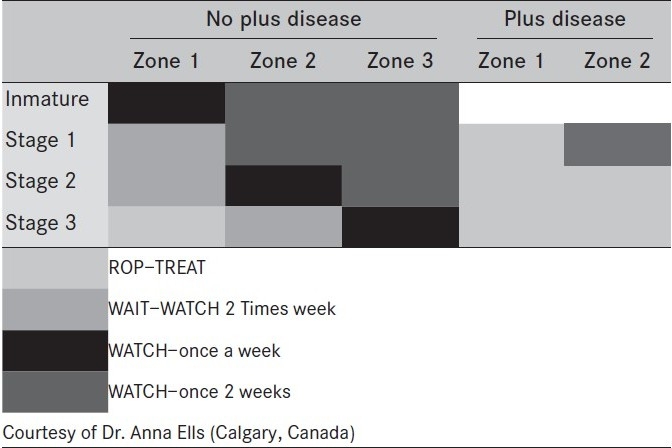
Recommended treatment guidelines according to PLUS Disease

## CONCLUSIONS

With our understanding of the pathophysiology of retinopathy of prematurity from the role of proangiogenic factors to the cascade of events ending with dilation and tortuosity of posterior vessels, it is possible to predict the natural history of ROP and its close relationship to salvaging the eye as the disease progresses. Plus disease has been described as a factor of prognostic significance in determining stage, diagnosis, and signs of severity of the disease. Our access to current technology and the availability of objective diagnostic tools may lead us to earlier recognition and appropriate treatment. The presence of plus disease has now become an indication of severity and for determining the adequate moment of treatment. Although zones and stages of ROP are noted, they are of secondary importance in determining whether laser treatment is needed.^42^
